# Stereotactic body radiotherapy for re-irradiation of lung cancer recurrence with lower biological effective doses

**DOI:** 10.1007/s13566-014-0175-2

**Published:** 2014-12-10

**Authors:** Nisha R. Patel, Rachelle Lanciano, Karna Sura, Jun Yang, John Lamond, Jing Feng, Michael Good, Ed J. Gracely, Lydia Komarnicky, Luther Brady

**Affiliations:** 1 Department of Radiation Oncology, Delaware County Memorial Hospital, Philadelphia CyberKnife Center, Havertown, PA USA; 2 Department of Radiation Oncology, Drexel University College of Medicine, Philadelphia, PA USA

**Keywords:** SBRT, Re-irradiation, Lung cancer, Recurrence, Radiation

## Abstract

**Objective:**

Few studies have evaluated re-irradiation of lung cancer recurrences with stereotactic body radiotherapy (SBRT). This study evaluates outcomes with SBRT re-irradiation for recurrent lung cancer.

**Methods:**

Two hundred and seventy-eight patients treated with SBRT for lung cancer were retrospectively reviewed. Of those, 26 patients with 29 tumors were re-irradiated with SBRT. Ninety percent of tumors received prior external beam irradiation and 10 % received prior SBRT. Previous median radiation dose was 61.2 Gy with a median 8-month interval from previous radiation. The median re-irradiation SBRT dose was 30 Gy (48 Gy_10_ biological effective dose (BED)). Endpoints evaluated included local control, overall survival, and progression-free survival.

**Results:**

Twenty-five of 29 tumors were evaluable for local control, with 27 tumors (93 %) considered in-field recurrences. In-field crude local control rate was 80 % (20/25) with 1 and 2-year actuarial rates of 78.6 and 65.5 %, respectively. One and 2-year actuarial survival rates were 52.3 and 37.0 %, respectively. One and 2-year actuarial progression-free survival rates were 56.7 and 37.0 %, respectively. Fifty-five percent of patients reported acute/chronic grades 1 and 2 toxicities. No grade 3 or higher toxicities were reported.

**Conclusion:**

Patients with recurrent lung cancer have limited options. SBRT re-irradiation is tolerable even after a median 61.2 Gy to the re-irradiation site. The lower BED used provided acceptable progression-free survival with low toxicity. Given the poor prognosis with current treatment options, new paradigms for re-treatment should include SBRT-re-irradiation as an adjunct to systemic therapy for in-field lung cancer recurrence.

## Introduction

Treatment for locally advanced lung cancer has improved since the 1990s from external beam radiation (EBRT) alone to concurrent chemotherapy and radiation providing survival benefit [1–5]. Nearly 70 % of patients diagnosed with lung cancer will receive EBRT as initial treatment [6]. Despite the use of concurrent chemotherapy with EBRT, local-regional relapse can occur in up to 50 % of patients [1]. Treatment options for lung recurrence remain limited. Fibrosis and decreased functional lung reserve after previous thoracic irradiation in the face of baseline pulmonary disease can limit surgical options. The use of external beam re-irradiation has demonstrated significant toxicity and limited overall survival [7–12]. Second-line salvage chemotherapy remains a standard alternative therapy. However, low response rates and short durable control reinforce the need for improved local treatment modalities [13].

Few series have reported treatment outcomes for stereotactic re-irradiation for lung cancer recurrence after previous EBRT/SBRT with local control rates from 52 to 92 % and low progression-free survival [14–18]. Nearly 50 % of patients experienced worsening dyspnea with 33 % grade 3 or higher toxicity after SBRT re-irradiation, with median biological effective dose (BED) of 100 Gy_10_ in one of the largest series to date [14]. Although delivery of BEDs of at least 100 Gy_10_ has demonstrated excellent local control rates and improved survival for treatment of early stage medically inoperable lung cancer, these doses in the re-irradiation setting may not be appropriate given the higher risk of associated toxicity [19–22]. This study reports a single institutional experience for re-irradiation with SBRT for in-field lung cancer recurrence and evaluates any apparent differences in outcome with the use of lower BEDs than used for primary treatment.

## Methods

The records of 278 consecutive patients treated with SBRT for lung cancer at Philadelphia CyberKnife, from January 2008 to December 2011, were retrospectively reviewed. Of those 278 patients, 26 patients with 29 tumors that received SBRT re-irradiation were further analyzed on this IRB-approved retrospective study. Twenty-six tumors previously received standard fractionated EBRT, and three tumors received SBRT prior to SBRT re-irradiation for lung recurrence. A minimum of 3-month follow-up with CT or PET/CT was required for evaluation of local control with 25 out of 29 tumors evaluable for local control. All patients were evaluable for survival. Local failure was defined as relapse within the SBRT PTV. Response evaluation criteria in solid tumors was used as a general guideline, and the use of PET/CT with SUV greater than or equal to pre-treatment values was considered a recurrence as previously reported [23]. Other patterns of failure including out-of-field thoracic progression and distant metastases were also evaluated by CT or PET/CT for progression-free survival analysis.

**Fig. 1:**
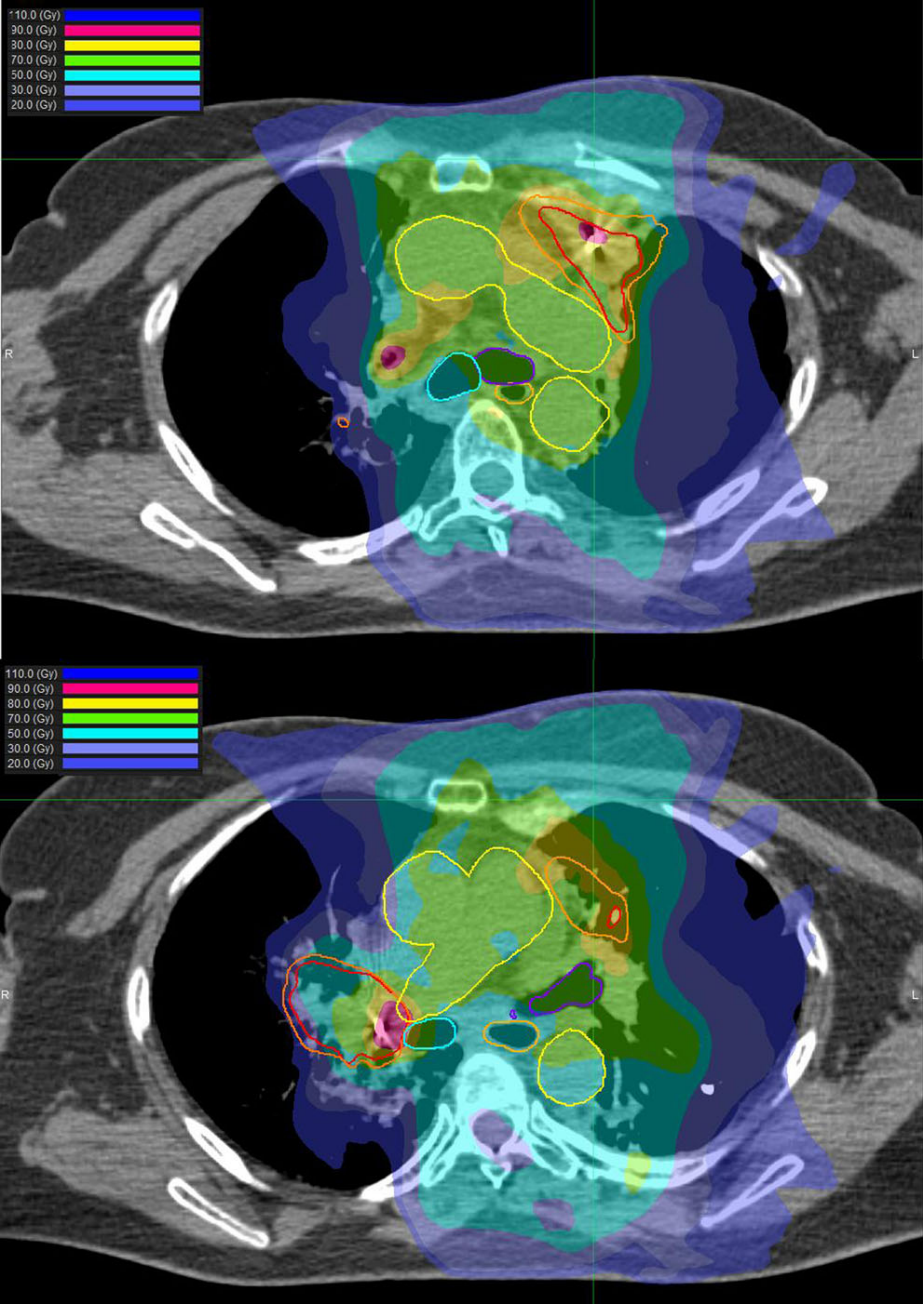
Composite plan of a patient who initially received 54 Gy external beam radiation for a left upper lobe adenocarcinoma. She was re-irradiated with SBRT 25 Gy in 5 fractions for an in-field recurrence in left upper lobe and subsequent 50 Gy in 5 fractions for a marginal right hilar recurrence. Note fiducials in each site of re-irradiation SBRT. Patient survived 45 months after initial SBRT re-irradiation

All patients were treated supine with the CyberKnife stereotactic system. Treatment planning included inspiration and expiration CT of chest to evaluate movement of the GTV with respiration. KV orthogonal imaging was used for real-time target tracking with either fiducial (*n* = 9), x-site lung (*n* = 3), or x-site spine (*n* = 17). Internal target volume was delineated for patients tracked with x-site spine. All clinical target volumes had an isotropic 5-mm expansion to create the planning target volume to account for inherent set up error/imaging lag time. Non-coplanar pencil beams using 6MV photons with ray-tracing dosimetric calculation for delivered dose were utilized for all treatment plans except one. Monte Carlo calculations were used for a single treatment plan; this option has since become the standard method of dose calculation within our institution. All treatment plans were evaluated to determine whether the re-treated SBRT lesion was considered an “in-field recurrence” of the previous radiation course as reported in other series [14] (Fig. 1). There were two tumors juxtaposed at the 50 % isodose line or field edge of previous external beam radiation plan that were considered marginal relapses with all other re-treated tumors considered to be in-field relapses. The Kaplan-Meier method was used to calculate the actuarial local control and overall survival rate. Statistical significance was evaluated by the log-rank test. Potential differences for local control between BED <48 and BED ≥48 Gy_10_ were also evaluated using the *χ* ^2^ test.

## Results

Patient characteristics are illustrated in Table 1. The median age was 68 years old (range 42–87). Of the 26 patients, 19 (73 %) were women and 7 (27 %) were men. The patients’ initial presentation at diagnosis was predominantly locally advanced non-small cell lung cancer (NSCLC) with 15 (58 %) patients stage III disease and 23 (88 %) non-small cell histology. The majority, 19 patients (73 %), previously received definitive external beam radiation therapy. Two patients (8 %) previously had postoperative radiation, two patients (8 %) previously received palliative radiation, and three patients (11 %) had prior SBRT. Initial chemotherapy was administered to 24 patients: concurrently with radiation in 21 (81 %) and adjuvantly in three patients (11 %).

**Table 1:** Patient characteristics

		Number	Percent
Sex	Female	19	73
	Male	7	27
Initial stage	I–II	8	31
	III	15	58
	IV	3	11
Initial histology	NSCL	23	88
	Non-NSCL	3	12
Type of initial RT	Definitive	19	73
	Post-op	2	8
	Palliative	2	8
	SBRT	3	11
Initial chemotherapy	Concurrent	21	81
	Adjuvant	3	12
	None	2	7

The median interval time from previous EBRT or SBRT to re-irradiation with SBRT was 8 months (range 3–26 months) (Table 2). Twenty-six tumors (90 %) had previous EBRT, and three tumors (10 %) had previous SBRT. A total of 27 tumors (93 %) were considered in-field recurrences receiving at least 30 Gy from previous radiation treatment with two marginal relapses. There were 17 peripheral tumors and 12 central tumors. The median previous dose of EBRT was 61.2 Gy (30–74 Gy) with three patients that had original diagnosis of stage IV disease accounting for previous lower palliative doses. Stable distant or intrathoracic metastatic disease was present in four patients at the time of re-irradiation with SBRT. After re-irradiation, 50 % (13 patients) received chemotherapy.

**Table 2:** Re-irradiation parameters

	Median	(Range)	BED <48 Gy (*n* = 12)	BED ≥48 Gy (*n* = 17)
Previous RT dose (Gy)	61.2	(30–74)	68.3	59.4
Time from prior RT (months)	8	(3–26)	7.5	8
Re-irradiation RT dose (Gy)	30	(15–50)	23	40
Re-irradiation no. of fractions	5	(3–5)	5	5
Re-irradiation BED dose (Gy)	48	(19.5–112.5)	36.6	72.2
Re-irradiation EQD2 dose (Gy)			30.5	60

**Fig. 2:**
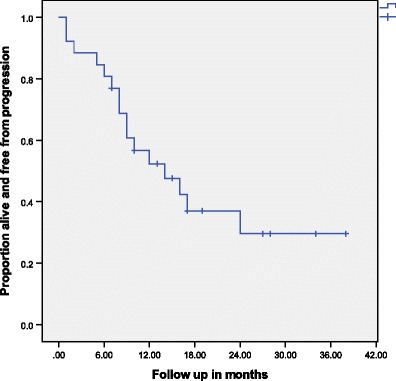
Proportion of patients free from progression over time

Median prescribed dose of SBRT for re-irradiation was 30 Gy (15–50, median BED of 48Gy_10_) delivered for a median of 5 fractions (range 3–5) prescribed to a median 69 % isodose line (range 55–85 %). The median tumor size treated was 3.2 cm (1.2–9.5 cm). Since the dose-per-fraction used in this study is <10Gy, and the LQ model is relatively accurate in this dose region (<10Gy/day), we adopted the LQ model to evaluate biological effective dose. Twelve tumors were treated with BED <48 Gy_10_ with a median BED dose of 36.6 Gy_10_ (9.5–42 Gy), and 17 tumors were treated with BED ≥48 Gy_10_ with a median BED dose of 72.2 Gy_10_ (48–112.5 Gy). The EQD2 doses for the two groups were 30.5 and 60 Gy, respectively. Twenty-five tumors were evaluable with radiographic assessment for 1 and 2-year actuarial local control rates. All 26 patients were evaluable for 1 and 2-year actuarial survival and progression-free survival rates. The median survival from SBRT re-irradiation was 14 months. The 1 and 2-year actuarial survival rates were 52.3 and 37.0 %, respectively, for the study sample. The 1 and 2-year actuarial progression-free survival rates were 56.7 % (95 % CI 37.3–76.1) and 37.0 % (95 % CI 16.8–57.2), respectively (Fig. 2), with no significant difference between BED <48 and BED ≥48 Gy_10_ (*p* = 0.76). The crude local control was 80 % (20/25) for the study sample. The 1 and 2-year actuarial local control rates were 78.6 % (95 % CI 60.0–97.2) and 65.5 % (95 % CI 37.5–93.5), respectively. All five local failures occurred within the PTV with three failures receiving BED <48 Gy_10_ and two failures receiving BED ≥48 Gy_10._ Further analysis demonstrated higher 1 year actuarial local control rates for BED ≥48 Gy_10_ of 90.9 % versus 62.5 % for BED <48Gy_10_; however, this difference was not statistically significant (*p* = 0.32). The durability of local control diminished over time with 2-year actuarial local control rate of 68.2 % for BED ≥48 Gy_10_ with a similar rate of 62.5 % for lower BED <48 Gy_10_. Further subset analysis did not demonstrate any statistically significant difference for local control stratified by peripheral or central location or time to re-irradiation with less than or greater than 12 months from initial radiation therapy.

No patients experienced grades 3–5 toxicity based upon the NCI CTCAE criteria without local thoracic or in-field progression of disease. One patient was hospitalized for progressive dyspnea with a large malignant pleural effusion and another patient underwent surgery for tracheoesophageal fistula with the presence of tumor cells noted within the fistula. Of the 29 treated lesions, 55 % experienced acute/late grades 1 and 2 symptoms with four reported symptoms within the dose range of BED <48 Gy_10_, and 12 reported symptoms for delivered doses of BED ≥48Gy_10._ Reported side effects included dyspnea, cough, fatigue, esophagitis, and skin rash (Table 3).

**Table 3:** Grades 1 and 2 toxicity

		BED <48 Gy	BED ≥48 Gy
Cough	Grade 1	0	2
	Grade 2	0	1
Pneumonitis	Grade 1	0	0
	Grade 2	0	1
Esophagitis	Grade 1	2	2
Skin	Grade 1	1	0
Fatigue	Grade 1	0	5
Dyspnea	Grade 1	1	1

## Discussion

Lung recurrence after definitive therapy poses a clinical management dilemma specifically for in-field lung cancer recurrences with relatively poor prognosis utilizing currently available treatment options. There have been few retrospective studies assessing the role of SBRT re-irradiation for lung recurrence; however, these studies include small numbers of patients with heterogeneous populations.

One of the largest series by Kelly et al. from M.D. Anderson reported 36 patients with a median time between EBRT and SBRT of 22.0 months. This study included a heterogeneous group with 31 % in-field recurrence, 36 % isolated out-of field recurrence, and 33 % recurrence in the setting of controlled disseminated disease [14]. The majority of patients were treated with 50 Gy in 4 fractions. Two-year intrathoracic relapse rate was 74 % despite excellent local control rates of 92 % [14]. On subset analysis, the patients treated for out-of-field recurrence had significantly longer progression-free survival (*p* = 0.04). Thus, a comparison to our series is difficult since we primarily report a worse prognostic group of in-field lung cancer recurrences, which constituted 93 % of the current series.

Peulen et al. also investigated the option of SBRT re-irradiation with a mean EQD2 dose of 109 Gy after prior SBRT for 32 lung tumors. Approximately 34 % of the patients had primary lung cancer recurrence while the majority of patients had re-irradiation for lung metastases from colorectal, renal cell carcinoma, or other primary sites. The primary endpoint for this study was toxicity. The patients (*n* = 9) experiencing grades 3–5 toxicity were found to have larger CTV volumes during initial SBRT, central tumors, and shorter median interval time to re-irradiation with SBRT (median 14.5 months) [15]. Of the 10 evaluated centrally treated recurrences in our study, there were no patients who had experienced grade 3 or higher toxicity. Although there were three patients within our study that also underwent re-irradiation with SBRT following initial SBRT, no patients experienced grades 4 or 5 toxicities. However, the improved toxicity profile in our study may be attributed to the lower SBRT re-irradiation doses in comparison to the above studies.

A small study reporting treatment outcomes specifically for lung cancer recurrences for SBRT re-irradiation after conventional EBRT was reported by Seung et al., who reported on eight patients treated with 40–60 Gy in 3 to 5 fractions with high local control rate of 86 % for lung cancer recurrence after prior EBRT (median follow-up 18 months). The study was limited by the small number of patients and did not explicitly specify whether the lesions were considered in-field or out-of-field recurrences [16].

Trakul et al. from Stanford also assessed treatment outcomes for SBRT re-irradiation for in-field recurrences after prior conventional irradiation [17]. However, this study included oligometastatic disease as well as lung cancer recurrences. The median BED was 80Gy for re-irradiation with SBRT that provided local control rates of 65.5 % at 12 months. Neither our study nor Trakul et al. reported any grade 2 or higher pneumonitis, unlike studies using higher re-irradiation doses [14–16]. Improved local control was also associated with longer interval time between re-treatment >16 months (*p* = 0.042).

A recent study by Trovo et al. reported the use of lower SBRT re-irradiation doses via tomotherapy (30 Gy in 5 to 6 fractions) to in-field recurrences after initial definitive therapy (*n* = 17) [18]. One-year local control rate was 86 %, and 1 and 2-year rates for overall survival were 59 and 29 %, respectively, with a predominant trend toward distant failure. Despite the median interval of 18 months prior to re-irradiation with SBRT and lower radiation doses, 4 out of 17 patients (23 %) experienced grade 3 toxicity or higher and two radiation-related deaths were reported. One patient had persistent hilar disease with fatal hemoptysis, so it is uncertain whether progression of disease may have been a contributing factor. In addition, all lesions were centrally located, posing a significant challenge to minimize potential toxicity to critical adjacent organs.

The current study evaluated treatment of in-field lung cancer recurrence with SBRT re-irradiation after conventional dose EBRT or SBRT. SBRT re-irradiation was delivered with a conservative dose of median BED of 48Gy_10._ There was no significant difference in 1 to 2-year actuarial local control between BED <48 Gy_10_ (EQD2 30.5 Gy) and BED ≥48 Gy_10_ (EQD2 60 Gy). The small number of patients in this study may have influenced this result, obscuring a true difference between the two groups. The 2-year actuarial local control of 65.5 % for the entire group was in close agreement with the Trakul et al. study, which reported 1-year actuarial local control of 65.5 %. Both this study and that of Trakul et al. have similar patient groups with previous conventional dose EBRT and re-irradiation with SBRT for in-field recurrence with similar local control, progression-free survival, and survival rates. The Kelly et al. study, which utilized higher dose re-irradiation with better local control rate, had similar 2-year actuarial progression-free survival rate to the current series. The poor 2-year actuarial progression-free survival of 37 % for our study and others validates the need to evaluate different paradigms for treating lung cancer recurrence with combination of targeted therapies and an appropriate SBRT re-irradiation dose. The possibility of intrathoracic and distant microscopic disease at the time of re-irradiation may prevent the benefit from higher local control rates with 100 Gy_10_ BED doses.

Potential toxicity also becomes a serious concern when considering re-irradiation. Without the potential benefit of progression-free survival, high local control rates may only add to increased toxicity and poor quality of life depending upon the location of the lesion. Several studies have suggested an interval of at least 5–6 months between re-treatment to mitigate possible toxicity to organs at risk such as the spinal cord [24, 25]. Despite the shorter median interval time for re-treatment of 8 months in this study, there were no patients that experienced grades 3–5 toxicity with the delivery of conservative radiation dose. In total, 55 % of patients had low-grade toxicity associated with re-irradiation, and the majority of reported symptoms occurred in the BED ≥48 Gy_10_ group. There was a single reported case of grade 2 pneumonitis in the study, which is lower than previous reports of nearly 7–55 % with the use of higher dose SBRT or EBRT re-irradiation and much lower than Kelly et al. report of 50 % grades 2 and 3 pneumonitis [7, 8, 14]. With real-time tracking and correction, we utilized PTV margins of 5 mm around GTV compared to the Kelly et al. method of utilizing an internal gross tumor volume (iGTV) with 11 mm margin around the iGTV. Larger margins around the GTV, higher dose delivered, and larger volumes of previously non-irradiated lung tissue may have contributed to their higher pneumonitis rates [14, 26].

In this study, progression of disease was the main cause of morbidity after SBRT re-irradiation. Despite treatment, actuarial 2-year progression-free survival of 37 % warrants new treatment approaches and enrollment in clinical trials. The only reported independent predictor for increased progression-free survival with re-irradiation has been the presence of isolated out-of-field recurrence suggestive of a metachronous lung primary [14]. Therefore, careful consideration of site and time to recurrence is important when deciding on the appropriate dose for re-irradiation. Despite improved outcome for re-irradiation of out-of-field recurrences, there is potential increased toxicity due to compromise of non-irradiated lung tissue. Dose escalation for SBRT re-irradiation may improve outcome for out of field metachronous primaries; however, this course should be undertaken cautiously. The inclusion of potential systemic targeted therapies and appropriate SBRT re-irradiation doses should be considered to benefit disease-free survival and quality of life for future treatment of in-field lung cancer recurrences due to the significant intrathoracic and distant failure rates.

## Conclusion

SBRT can be considered an option for patients with lung cancer recurrence after high dose definitive radiation with concurrent systemic therapy. Dose of SBRT re-irradiation should be individualized after review of the patient’s potential risk for toxicity and the possibility of curative approach to optimize the quality of life for these poor prognostic patients.
